# Supplementary material for: A Multilayer Functionalized Drug-Eluting Balloon for Treatment of Coronary Artery Disease
Source: Pharmaceutics. 2021 Apr 23;13(5):614. doi: 10.3390/pharmaceutics13050614 (PMC8146216; doi:10.3390/pharmaceutics13050614)
Supplement: Supplementary file 1 [file pharmaceutics-13-00614-s001.zip › pharmaceutics-1185676-supplementary.pdf]

pharmaceutics

Supplementary Materials: A Multilayer Functionalized Drug-Eluting Balloon for Treatment of Coronary Artery Disease

Hak-Il Lee, Won-Kyu Rhim, Eun Young Kang, Bogyu Choi, Jun Hyeok Kim and Dong Keun Han

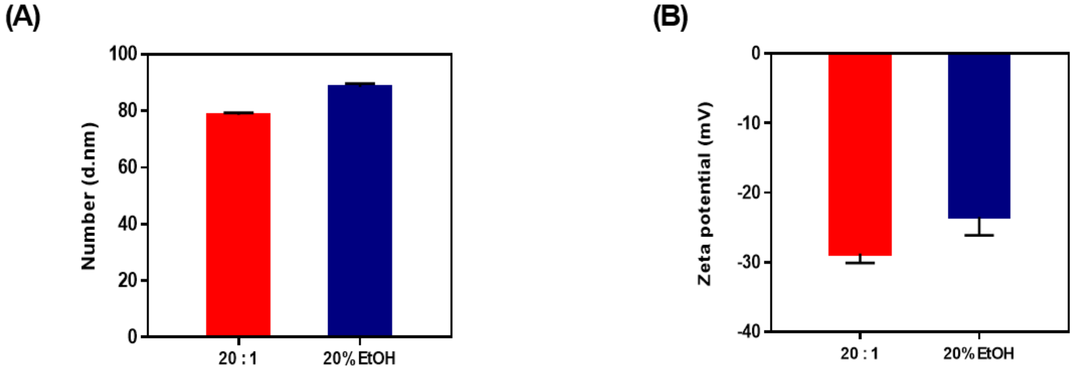

Figure S1. Characterization of liposome. (**A**) Size and (**B**) Zeta potential of optimized ratio of EVL-loaded liposome and EVL-loaded liposome rehydrated with 20% ethanol. Values are presented as mean ± SD (n = 3).

Citation: Lee, H.-I; Rhim, W.-K.; Kang, E.Y.; Choi, B.; Kim, J.H.; Han, D.K. A Multilayer Functionalized Drug-Eluting Balloon for Treatment of Coronary Artery Disease. *Pharmaceutics* **2021**, *13*, 614. https://doi.org/10.3390/pharmaceutics13050614

Academic Editor: Sophia G. Antimisiaris

Received: 31 March 2021
Accepted: 19 April 2021
Published: 23 April 2021

Publisher's Note: MDPI stays neutral with regard to jurisdictional claims in published maps and institutional affiliations.

Copyright: © 2021 by the authors. Licensee MDPI, Basel, Switzerland. This article is an open access article distributed under the terms and conditions of the Creative Commons Attribution (CC BY) license (http://creativecommons.org/licenses/by/4.0/).
